# Mechanisms of pro-arrhythmic abnormalities in ventricular repolarisation and anti-arrhythmic therapies in human hypertrophic cardiomyopathy

**DOI:** 10.1016/j.yjmcc.2015.09.003

**Published:** 2016-07

**Authors:** Elisa Passini, Ana Mincholé, Raffaele Coppini, Elisabetta Cerbai, Blanca Rodriguez, Stefano Severi, Alfonso Bueno-Orovio

**Affiliations:** a Department of Computer Science, University of Oxford, Oxford OX13QD, United Kingdom; b Department of Electrical, Electronic and Information Engineering, University of Bologna, Cesena 47521, Italy; c Department NEUROFARBA, University of Florence, Florence 50139, Italy

**Keywords:** Hypertrophic cardiomyopathy, Pro-arrhythmic mechanisms, Repolarization reserve, Inter-subject variability, In silico drug testing

## Abstract

**Introduction:**

Hypertrophic cardiomyopathy (HCM) is a cause of sudden arrhythmic death, but the understanding of its pro-arrhythmic mechanisms and an effective pharmacological treatment are lacking. HCM electrophysiological remodelling includes both increased inward and reduced outward currents, but their role in promoting repolarisation abnormalities remains unknown. The goal of this study is to identify key ionic mechanisms driving repolarisation abnormalities in human HCM, and to evaluate anti-arrhythmic effects of single and multichannel inward current blocks.

**Methods:**

Experimental ionic current, action potential (AP) and Ca^2 +^-transient (CaT) recordings were used to construct populations of human non-diseased and HCM AP models (n = 9118), accounting for inter-subject variability. Simulations were conducted for several degrees of selective and combined inward current block.

**Results:**

Simulated HCM cardiomyocytes exhibited prolonged AP and CaT, diastolic Ca^2 +^ overload and decreased CaT amplitude, in agreement with experiments. Repolarisation abnormalities in HCM models were consistently driven by L-type Ca^2 +^ current (I_CaL_) re-activation, and I_CaL_ block was the most effective intervention to normalise repolarisation and diastolic Ca^2 +^, but compromised CaT amplitude. Late Na^+^ current (I_NaL_) block partially abolished repolarisation abnormalities, with small impact on CaT. Na^+^/Ca^2 +^ exchanger (I_NCX_) block effectively restored repolarisation and CaT amplitude, but increased Ca^2 +^ overload. Multichannel block increased efficacy in normalising repolarisation, AP biomarkers and CaT amplitude compared to selective block.

**Conclusions:**

Experimentally-calibrated populations of human AP models identify I_CaL_ re-activation as the key mechanism for repolarisation abnormalities in HCM, and combined I_NCX_, I_NaL_ and I_CaL_ block as effective anti-arrhythmic therapies also able to partially reverse the HCM electrophysiological phenotype.

## Highlights

• In silico populations of human ventricular AP models recapitulate the HCM phenotype.
• HCM remodelling enlarges variability and promotes repolarisation abnormalities (EADs).
• I_CaL_ re-activation underlies repolarisation abnormalities in human HCM.
• Selective inward current blocks suppress EADs and partially reverse the HCM phenotype.
• I_NCX_ block improves the efficacy of I_NaL_ and I_CaL_ selective blocks in HCM.

## 1 Introduction

Hypertrophic cardiomyopathy (HCM) is the most common monogenic cardiac disorder and the main cause of sudden cardiac death in children and young adults [1], with a reported prevalence of 1 in 500 worldwide [2]. Usually asymptomatic, it is characterised by an unexplained thickening (hypertrophy) of the left ventricle, and occasionally of the right, with predominant involvement of the inter-ventricular septum. However, the ejection fraction is usually preserved in HCM patients [3], [4], with only a minority of the subjects developing enlarged ventricular cavities, hence pointing towards a different aetiology of the disease compared to acquired heart failure. In addition, the first manifestation of HCM is often arrhythmic sudden death, caused by ventricular tachyarrhythmias [2], but the underlying electrophysiological mechanisms remain unclear.

HCM is still lacking of a disease-specific pharmacological treatment [5], [6], [7]. To date, implantable cardioverter-defibrillator therapy prevails as the only effective prevention of sudden cardiac death in HCM [8], [9]. Different mutations are associated with different outcome in HCM patients, but the strength of the genotype-phenotype correlation is weak to warrant recommendation in risk management based on the genotype, due to significant inter-subject variability in disease expression, even among carriers of the same variant [10]. Therefore, a better understanding of the ionic mechanisms underlying arrhythmic risk and inter-subject variability in HCM is required to guide the development of specific pharmacological treatments and risk stratification.

Recently, Coppini et al. characterised the electrophysiological profile of human HCM by measuring alterations in the action potential (AP), Ca^2 +^ subsystem, sarcolemmal ionic currents and mRNA expression in donor HCM cardiomyocytes [3]. Particularly distinct to the ionic remodelling associated to heart failure [11], HCM cardiomyocytes exhibited a significant overexpression of the L-type Ca^2 +^ current (I_CaL_), which together with an increase in the Late Na^+^ (I_NaL_) and a reduction in K^+^ repolarising currents, contributed to a prolonged AP and Ca^2 +^ transient (CaT). Experimental recordings also provided evidence of HCM facilitating the occurrence of repolarisation abnormalities such as early after-depolarisations (EADs), which may act as triggers for ventricular arrhythmias [12], [13].

Repolarisation abnormalities and in particular EADs are known to be facilitated by abnormalities in the Ca^2 +^ subsystem and Ca^2 +^ overload [14], [15], [16]. Genetic mutations in HCM commonly enhance Ca^2 +^-sensitivity and energy requirements of myosin ATPase, leading to altered force production and impairment of intracellular Ca^2 +^ and intracellular Ca^2 +^ load [6], [8]. Indeed, experimental data in human HCM provided evidence of a highly impaired Ca^2 +^ subsystem, owing to a reduction of Ca^2 +^ uptake by SERCA and an altered Na^+^/Ca^2 +^ exchanger function, contributing to the slower kinetics of CaT and the elevated diastolic Ca^2 +^ [3]. Further investigations are therefore required to advance our understanding of the ionic mechanisms underlying pro-arrhythmic repolarisation abnormalities in HCM.

The goals of this study are to investigate the ionic mechanisms driving repolarisation abnormalities in human HCM cardiomyocytes, and to evaluate the efficacy of selective and combined inward currents block as potential anti-arrhythmic strategy. Building on the comprehensive dataset provided by Coppini et al. [3] and on the methodology proposed by Britton et al. [17], we construct two populations of human ventricular AP models in range with the experimental data and accounting for cell-to-cell variability in non-diseased and HCM conditions. Based on our analysis in these populations, we propose different single and multi-channel strategies for the pharmacological management of cellular repolarisation abnormalities in HCM, evaluating their efficacy in abolishing repolarisation abnormalities and in reversing the electrophysiological phenotypic characteristics of human HCM cardiomyocytes.

## 2 Materials and methods

### 2.1 Experimental data

The experimental dataset, previously published in [3], consists of recordings from n = 80 HCM cells and n = 31 non-failing non-hypertrophic control cells (CTRL). HCM cardiomyocytes were hypertrophic, as indicated by an increased cell volume (+ 90%) compared with CTRL (33.5 ± 4.3 vs 17.6 ± 3.2 pL, P < 0.05). Single cell patch-clamp measurements and intracellular Ca^2 +^ studies produced an extensive set of AP and Ca^2 +^-transient (CaT) biomarkers at 1 Hz pacing: AP duration (APD), computed at 20%, 50% and 90% of repolarisation (APD_20_, APD_50_ and APD_90_, respectively), AP amplitude (AP_amp_), mean upstroke velocity (dV/dt_MEAN_, computed as the mean dV/dt value during the upstroke phase), resting membrane potential (RMP), CaT time to peak (CaT_ttp_), CaT relaxation time from peak, computed at 50% and 90% of CaT decay (T_50_ and T_90_), CaT amplitude (CaT_amp_) and diastolic Ca^2 +^ concentration ([Ca^2 +^]_i,dias_). In addition, voltage clamp experiments, together with mRNA and protein expression studies, were considered.

### 2.2 Baseline model

As baseline for our investigations, the endocardial version of the O'Hara-Rudy (ORd) model was used [18]. This constitutes the most sophisticated human ventricular AP model to date, developed from and extensively validated against experimental data from more than 100 non-diseased human hearts. Minor modifications were performed to the original model in order to better reproduce the experimental non-diseased data considered in this study. A detailed description of these changes is provided in the extended methods and Supplemental Table S1.

### 2.3 Building the control population

As in Britton et al. [17], a population of non-diseased AP models accounting for biological variability was constructed, by assuming that variability is mostly caused by cell-to-cell differences in ion channel density rather than kinetics (which may be altered instead by abnormalities such as channelopathies [19]).

An initial population of 30,000 human endocardial AP models was generated, by varying a total of 11 parameters in the original model. These included the maximal conductances (g) of the main ionic currents/pumps/exchangers characterising the human ventricular AP: g_Kr_, g_Ks_, g_K1_, g_to_, g_CaL_, g_NaL_, g_Na_, g_NCX_, g_NaK_ (Na^+^/K^+^ pump), g_Rel_ (RyR) and g_Up_ (SERCA). All the parameters were probabilistically sampled in the [50%-150%] range with respect to their original values, by using Latin Hypercube Sampling [20].

From this initial pool of 30,000 candidate models, a calibration process was performed to select those models in agreement with the experimental non-diseased data [17]. Calibration ranges were extracted from each of the experimental AP and CaT biomarkers, by considering their minimum and maximum values. Only models within these experimental bounds, i.e. satisfying all the experimental constraints, were accepted in the final CTRL population, while the others were discarded.

Absolute [Ca^2 +^]_i,dias_ values were not used within the calibration process, since these are extracted from the conversion of fluorescence emission after removal of the background level signal [21]. Cell-to-cell offsets in background emission can hence significantly affect the indirect estimation of [Ca^2 +^]_i,dias_, whereas other CaT biomarkers such as CaT amplitude and duration are insensitive to diastolic Ca^2 +^ concentrations. We therefore reserved [Ca^2 +^]_i,dias_ data to compare diastolic Ca^2 +^ levels in HCM vs CTRL cardiomyocytes, by normalising them with respect to the mean CTRL values.

### 2.4 Building the HCM population

Based on the experimental data described above, we constructed the human HCM population by applying the electrical remodelling measured in human HCM to the CTRL population. Remodelling in the different ionic currents was accounted for by scaling their corresponding conductances, based on the ratio between HCM and non-diseased data, as reported by Coppini et al. [3]. This is equivalent to shifting in mean values the distributions of peak intensities for the different affected ionic currents, as experimentally reported. Based on the voltage clamp experiments, we up-regulated I_NaL_ (+ 165%) and I_CaL_ (+ 40%), and down-regulated I_to_ (− 70%) and I_K1_ (− 30%), together with an increase of the fast and slow time constants of both voltage- and Ca^2 +^-dependent I_CaL_ inactivation (+ 35% and + 20%, fast and slow respectively). Based on mRNA expression data, we modulated the K^+^ repolarizing currents (I_Kr_ and I_Ks_, − 45%), SERCA pump (J_up_, − 25%), RyRs release (J_rel_, − 20%) and Na^+^/Ca^2 +^ exchanger (I_NCX_, + 30%). Finally, we modified cell radius to reproduce the + 90% increase in cell volume reported in the experiments. In the absence of specific ultrastructural analysis of the human HCM cardiomyocytes, we assumed an equal volume increase of all subcellular compartments, in agreement with the increased cell radius and marked enlargement of the SR in murine models of HCM [22].

Three additional remodelling elements were considered: i) an increased affinity of Troponin for Ca^2 +^ (K_TRPN,_ − 50%) [23], [24]; ii) Na^+^/K^+^ pump inhibition due to energy deficiency of ATP-consuming processes (I_NaK_, − 30%) [24], [25], which markedly regulates intracellular Ca^2 +^ load by Na^+^ regulation of the Na^+^/Ca^2 +^ exchanger [26]; and iii) an increase of background Na^+^ current (I_Nab_, + 165% as for I_NaL_) [27]. A sensitivity analysis of model biomarkers for all the ionic parameters sampled in the CTRL and HCM human populations is provided in Supplemental Figure S2. For comparative purposes, the main differences in ionic remodelling between HCM and heart failure are highlighted in Supplemental Table S2.

### 2.5 Repolarisation abnormalities

All AP traces were automatically checked for repolarisation abnormalities, e.g. EADs. HCM models with abnormal repolarisation were identified, based on the following conditions: i) failure of repolarisation at the end of diastole (define as V_m_ > − 65 mV); and/or ii) showing a positive derivative of transmembrane voltage after 150 ms from AP peak. The final trace of models exhibiting repolarisation abnormalities was recorded for a full extent of 3 s. In accordance with experiments, these were subdivided into single/multiple EADs (based on the number of voltage peaks after the AP upstroke, for EADs lasting less than 3 s), and repolarisation failure (RF; EADs longer than 3 s). This latter group of abnormalities is also in agreement with independent experimental observations of long-lasting membrane potential oscillations after EADs onset due to slow repolarisation [28]. Matching experiments, models showing repolarisation abnormalities were not considered in the computation of mean AP and CaT biomarkers.

### 2.6 Current blocks

Based on our analysis of ionic mechanisms underlying repolarisation abnormalities in human HCM models, *in silico* studies were conducted to investigate the effects of inhibition of inward currents during the phase 2 of the human AP (I_NaL_, I_CaL_ and I_NCX_). This was achieved by reducing their respective maximal ion channel conductances, for all models within the HCM population. Single and multi-channel strategies, from 10% to 60% current block, were considered. Larger current blocks were not considered in order to avoid secondary effects due to almost complete repression of the respective ionic currents.

### 2.7 Simulation details

All models were paced at 1 Hz until steady state (500 s), to ensure that intracellular Na^+^ and K^+^ concentrations were stable over time. As in the experiments, the last ten AP and CaT traces for each model were stored and used to compute average AP and CaT biomarkers. Numerical simulations and biomarkers evaluation were performed using Chaste [29]. Post-processing of AP and CaT traces, together with data analysis, were performed in Matlab (Mathworks, Inc.).

## 3 Results

### 3.1 The ionic remodelling and abnormal Ca^2 +^ handling recover the main hallmarks of the human HCM electrophysiological phenotype

**Fig. 1:**
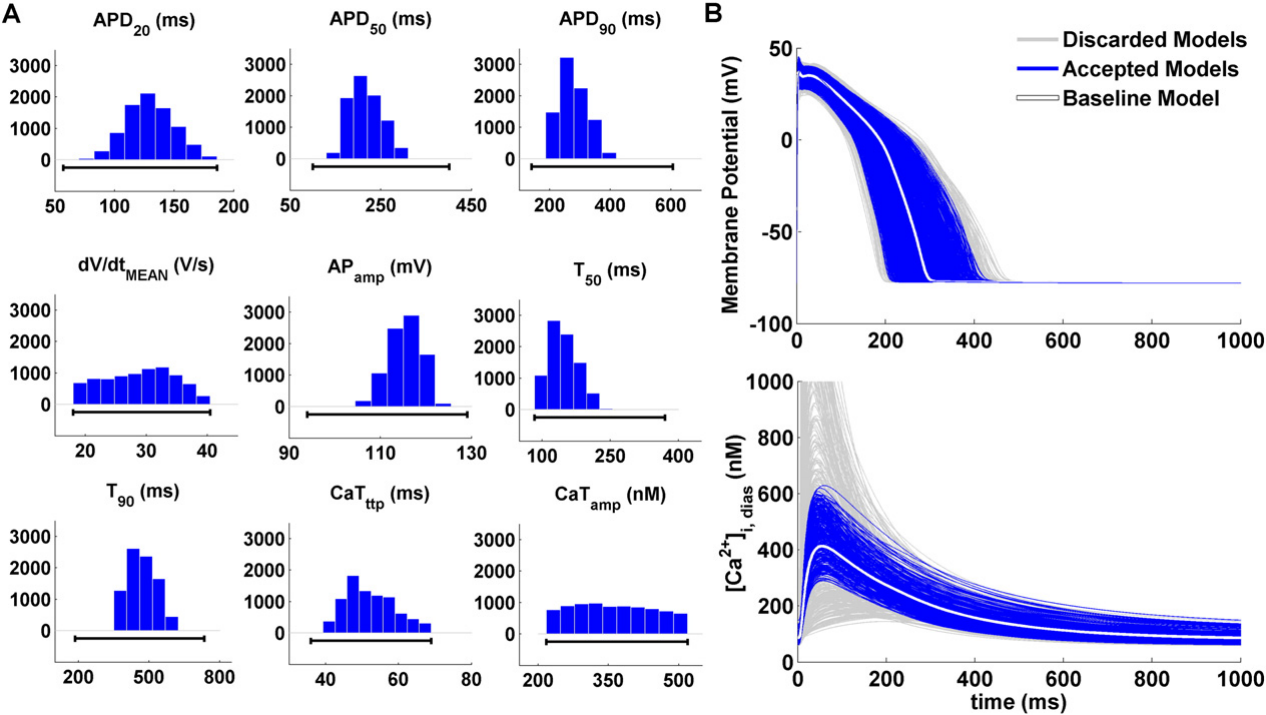
Experimentally-calibrated population of human ventricular non-diseased cell models. A: AP and CaT biomarker distributions for the 9118 models accepted in the CTRL population, all in agreement with the experimental ranges (black lines). Histograms show the number of models for each of the bins. B: Representative AP and CaT traces for cell models accepted in the final CTRL population (blue) and discarded ones (light grey). The baseline model traces have been highlighted in white.

As a result of the experimentally-driven calibration, the human ventricular CTRL population consists of 9118 models out of the initial pool of 30,000, qualitatively and quantitatively in agreement with the experimental recordings in human non-diseased cells, and accounting for biological variability. Fig. 1A illustrates the distribution of AP and CaT biomarkers in the non-diseased population: only models for which all AP and CaT biomarkers were within the experimental bounds were accepted in the final population, while others were discarded. Fig. 1B shows representative AP and CaT traces for both accepted (blue) and discarded (light grey) models. The baseline model used to generate the population has been highlighted in white.

**Fig. 2:**
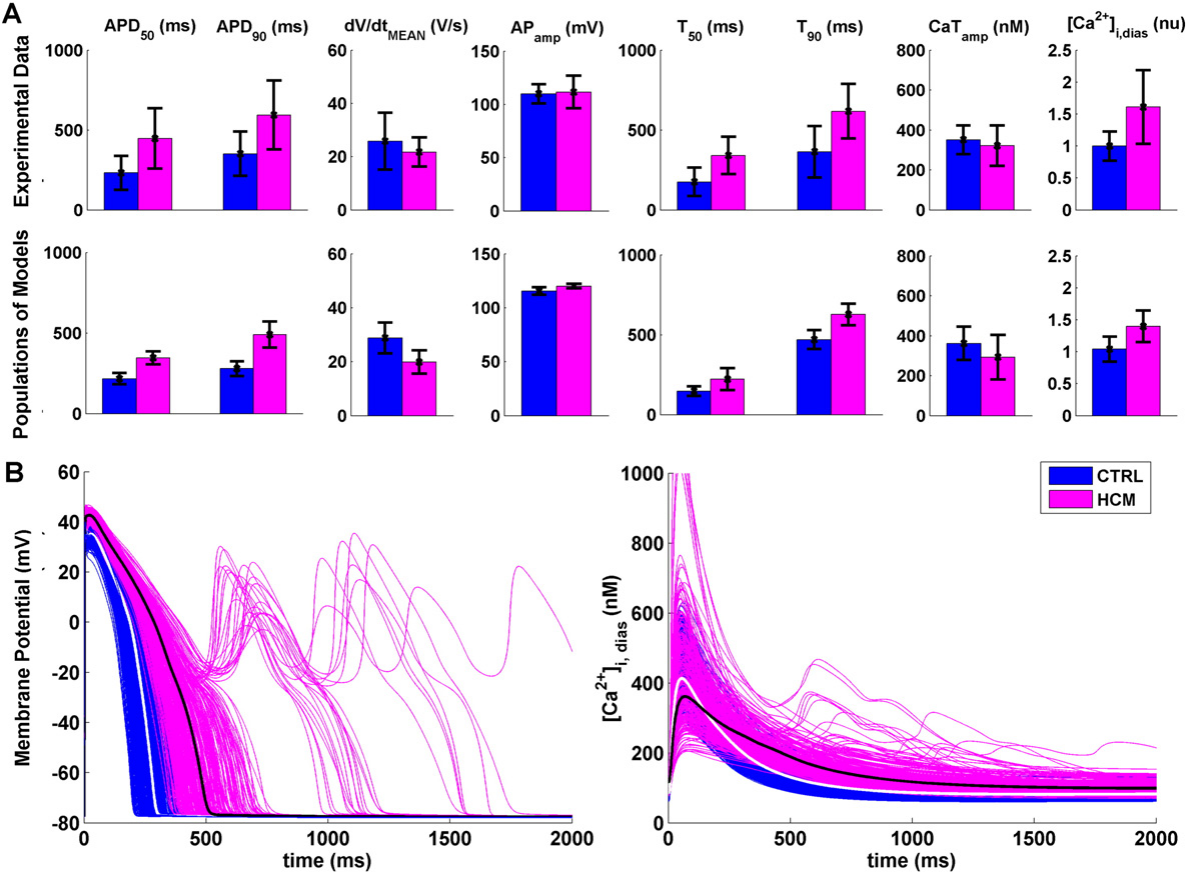
Comparison of the HCM population of human ventricular cell models (pink) vs CTRL (blue). A: Bar plots show AP and CaT biomarkers distribution in HCM *vs* CTRL for experiments (first row; AP biomarkers: n = 19 for CTRL, n = 57 for HCM; CaT biomarkers: n = 12 for CTRL, n = 22 for HCM) and populations of models (second row, n = 8366). Data are presented as mean ± SD. B: Representative AP and CaT traces of HCM and CTRL models. The action potential for the baseline CTRL and HCM models have been highlighted in white and black, respectively.

By applying the experimentally-recorded remodelling in HCM to the CTRL population (see Methods), the human HCM population was obtained. This population qualitatively and quantitatively reproduces the HCM electrophysiological phenotype, as shown by the AP and CaT biomarkers comparison in Fig. 2A. In agreement with the experiments, HCM models are characterised by prolonged AP, reduced upstroke velocity and slightly increased AP_amp_. CaT is prolonged as well, together with a decrease in CaT_amp_ and an increase in [Ca^2 +^]_i,dias_.

The main differences between HCM (pink) and CTRL (blue) are summarised in Fig. 2B, showing representative AP and CaT traces from the two populations. To facilitate the comparison, the baseline CTRL model and its corresponding HCM counterpart have been highlighted in white and black, respectively. Within the HCM population, approximately 8% of the models exhibited repolarisation abnormalities. As in the experiments, AP and CaT biomarkers of these models were not considered for the biomarkers comparison presented above, and are analysed in detail below.

### 3.2 I_CaL_ re-activation underlies repolarisation abnormalities in human HCM

**Fig. 3:**
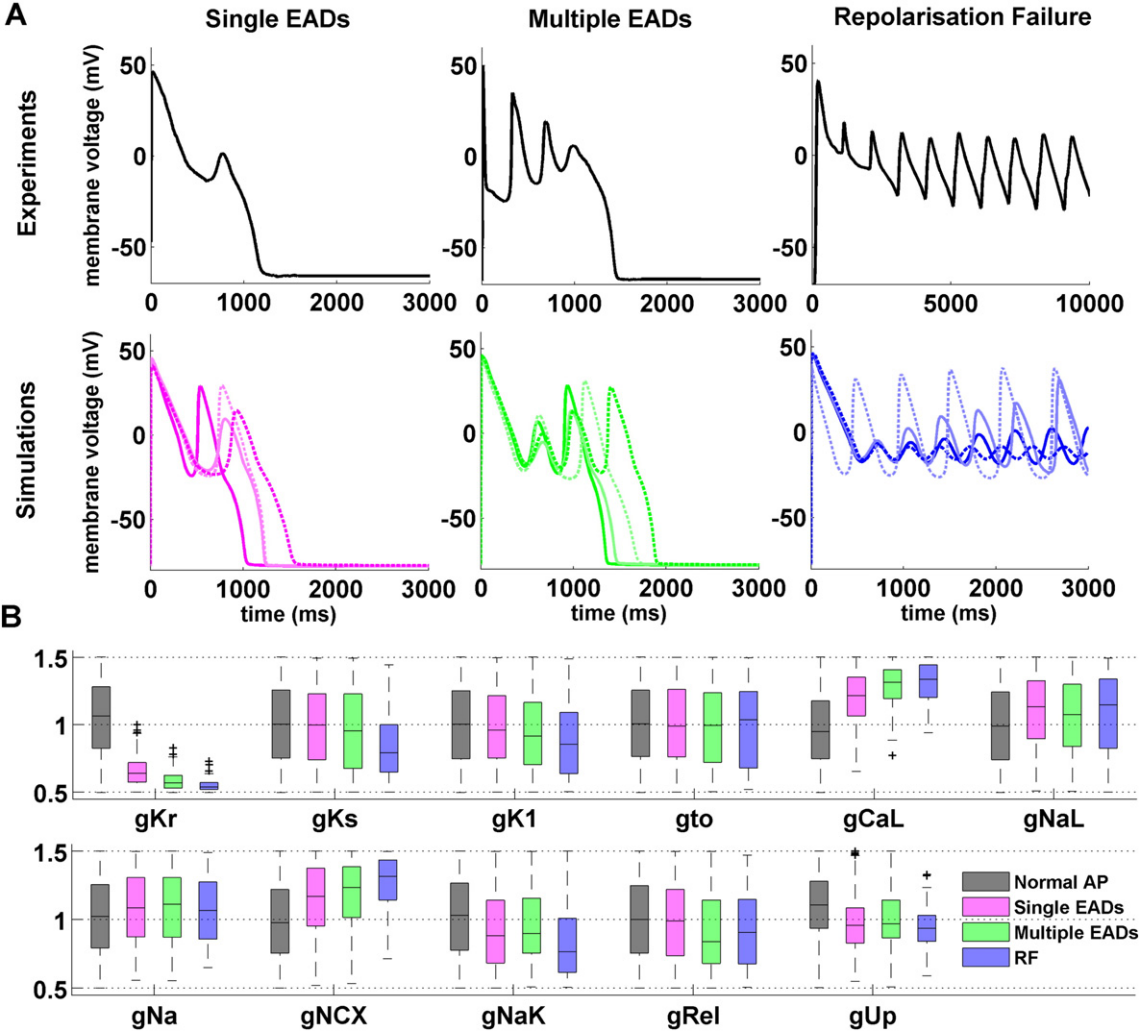
Repolarisation abnormalities in human HCM models (n = 752). A: Representative experimental (top) and simulated (bottom) HCM action potential traces, showing different types of repolarisation abnormalities. From left to right: single EADs, multiple EADs and repolarisation failure (RF). B: Normalised distributions of ionic properties for the 11 conductances varied within the population, for models displaying normal AP (n = 8366), single/multiple EADs (n = 480/201) and RF (n = 71). On each box, the central mark is the median, box limits are the 25th and 75th percentiles, and whiskers extend to the most extreme data points not considered outliers, plotted individually as separate crosses.

Repolarisation abnormalities were detected in 752 out of 9118 HCM models, and classified as single/multiple EADs (480 and 201 models, respectively) and repolarisation failure (RF, 71 models). Representative experimental and *in silico* AP traces from each of these subgroups are presented in Fig. 3A, highlighting the similarities between simulations and experimental recordings. Fig. 3B shows the distributions of ionic properties for each of the HCM repolarisation abnormalities subgroups, compared to the models with a normal AP, i.e. exhibiting a regular repolarisation phase. All models affected by repolarisation abnormalities were characterised by markedly low I_Kr_ conductances. In these subgroups, models were also characterised by decreasing conductances of I_Ks_, I_K1_ and I_NaK_ currents, which further contributed to compromise the repolarisation reserve [30]. In addition, all inward currents during the phase 2 of the AP (I_CaL_, I_NaL_ and I_NCX_) were up-regulated in HCM models displaying repolarisation abnormalities. Finally, Ca^2 +^ uptake through SERCA and Ca^2 +^ release through RyRs were moderately reduced compared to HCM models with normal repolarisation.

**Fig. 4:**
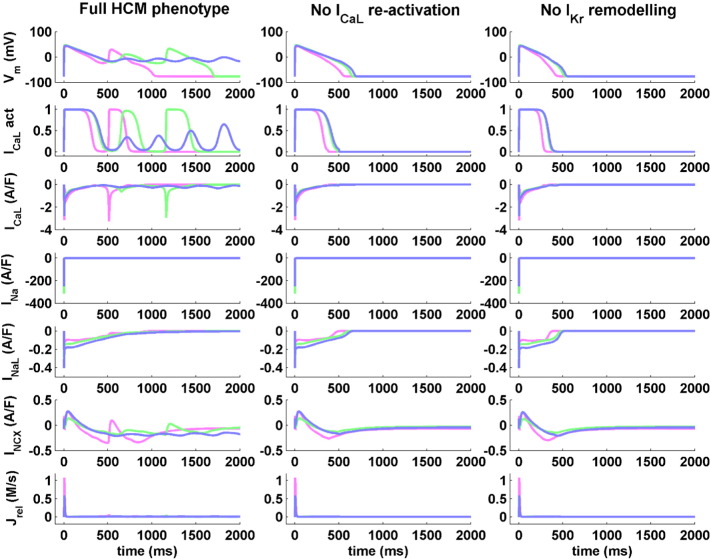
I_CaL_ re-activation underlies repolarisation abnormalities in human HCM models. From top to bottom, representative traces for AP, I_CaL_ activation, transmembrane inward currents (I_CaL_, I_Na_, I_NaL_ and I_NCX_) and Ca^2 +^ release from the sarcoplasmic reticulum (J_rel_) are shown (left column). Selected HCM models are presented from the three repolarisation abnormalities subgroups: single EADs (pink), multiple EADs (green) and RF (blue). Simulation results are shown for the same models when inhibiting L-type Ca^2 +^ channels re-opening (middle column), as well as when eliminating the I_Kr_ remodelling induced by HCM (right column). In both cases, repolarisation abnormalities are completely abolished.

Fig. 4 further illustrates the ionic mechanisms underlying repolarisation abnormalities in HCM models. Single/multiple EADs and RF were consistently led by I_CaL_ re-activation in all 752 abnormal HCM models (Fig. 4, left column). The compromised repolarisation reserve kept the membrane depolarised long enough to allow for the re-opening of the L-type Ca^2 +^ channels, as confirmed by the I_CaL_ activation gate traces (Fig. 4, left column, second row). Simulation results did not identify any re-activation of the Na^+^ gates or any significant release of Ca^2 +^ from the sarcoplasmic reticulum (Fig. 4, left column, fourth and last rows).

In order to corroborate these findings, additional simulations were performed artificially impeding the re-activation of L-type Ca^2 +^ channels. This consistently eliminated all repolarisation abnormality types in all 752 abnormal HCM models (Fig. 4, middle column). Finally, to confirm the role played by the weak repolarisation reserve in leading to I_CaL_ re-activation, we considered additional simulation results obtained when eliminating the I_Kr_ remodelling associated to the human HCM phenotype. This again abolished all the observed repolarisation abnormalities within the remodelled HCM population (Fig. 4, right column).

### 3.3 Selective block of inward currents as a potential therapy in human HCM

Based on the analysis of the ionic mechanisms underlying repolarisation abnormalities in human HCM, we identified the selective blockade of the three main inward currents during the AP plateau phase (I_CaL_, I_NaL_ and I_NCX_) as potential anti-arrhythmic strategies in this disease. Indeed, the HCM models exhibiting repolarisation abnormalities were characterised by high conductances for these inward currents. This contributed to further reduce the repolarisation reserve, already compromised as a consequence of the reduced I_Kr_ associated to HCM remodelling, leading to a large APD prolongation and thus favouring I_CaL_ re-activation.

**Fig. 5:**
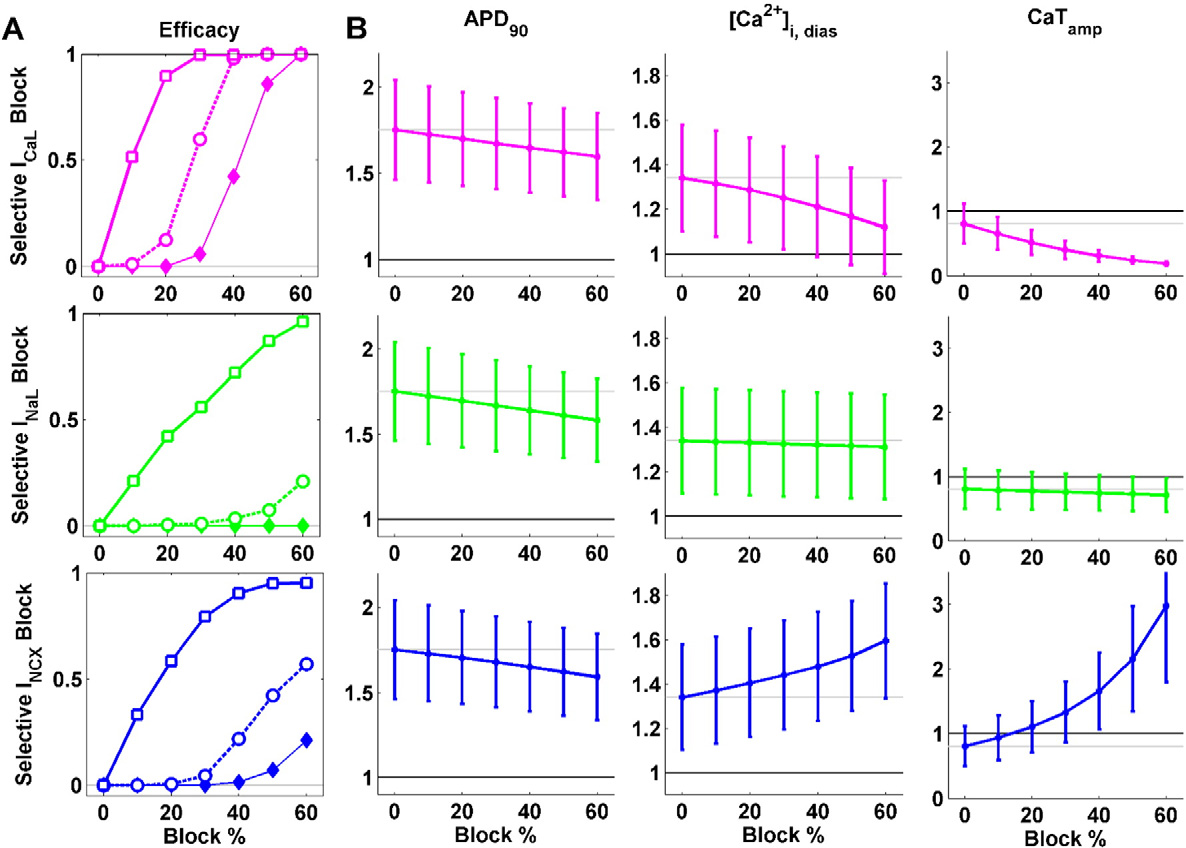
*In silico* evaluation of selective inward current block in human HCM. Each row shows the effect of a single current block: I_CaL_ (top, magenta), I_NaL_ (middle, green) and I_NCX_ (bottom, blue). A: Anti-arrhythmic efficacy of single channel blocks, evaluated as the fraction of repolarisation abnormalities successfully suppressed in the human HCM population. The efficacy is shown separately for each subgroup of repolarisation abnormalities: single EADs (thick solid, white squares), multiple EADs (thick dashed, white circles) and RF (thin solid, full diamonds). B: Effects of blocking each ionic current in reversing APD_90_, [Ca^2 +^]_i,dias_ and CaT_amp_ changes in the human HCM phenotype. Data are presented as mean ± SD, normalised with respect to the corresponding CTRL means. CTRL and HCM mean values are shown as black and grey lines, respectively, to facilitate comparison.

Simulation studies were conducted to investigate the effect of the selective block of these currents. Their therapeutic potency was evaluated by monitoring the abolishment of repolarisation abnormalities in the human HCM population, as well as their capability of normalising AP and CaT biomarkers in the rest of the HCM population (Fig. 5A and B, respectively).

The three current blocks were effective in reducing repolarisation abnormalities, especially single EADs (Fig. 5A, solid lines). Their efficacy was however lower in suppressing abnormalities under conditions of a highly compromised repolarisation reserve (Fig. 5A, dashed and thin solid lines for multiple EADs and RF, respectively). I_CaL_ block proved as the most effective option: indeed, 30% of I_CaL_ block sufficed to suppress all single EADs, and with a 60% I_CaL_ block all repolarisation abnormalities are abolished. I_NaL_ block significantly reduced the occurrence of single EADs (more than 95% with 60% block), but its effect was smaller for multiple EADs (20%) and null for RF. The efficacy of I_NCX_ block was higher than the one of I_NaL_, succeeding in suppress more than 95% of single EADs as well, but up to 60% of multiple EADs and 20% of RF instances. None of the three selective inward current blocks elicited additional repolarisation abnormalities in the rest of the human HCM population.

Regarding the normalisation of AP biomarkers, all the selective current blocks partially reversed the AP prolongation occurring in HCM cardiomyocytes (Fig. 5B, first column). The magnitude of APD reduction relative to the HCM population was similar for the three considered current blocks (i.e., APD_90_ decrease of − 7.8%, − 9.2% and − 8.6% for 60% of I_CaL_, I_NaL_ and I_NCX_ block, respectively).

Both I_CaL_ and I_NaL_ blocks reduced [Ca^2 +^]_i,dias_ (Fig. 5B, second column), one of the hallmarks induced by HCM remodelling. However, I_CaL_ blockade yielded a more remarkable reduction in diastolic Ca^2 +^ load (− 19.3% with 60% I_CaL_ block) than I_NaL_, which had a smaller effect (− 1.8% with 60% I_NaL_ block). On the contrary, blocking I_NCX_ led to an increase in [Ca^2 +^]_i,dias_ (+ 27% with 60% I_NCX_ block), thus potentially aggravating the HCM phenotype. A similar trend was observed for the CaT amplitude (Fig. 5B, third column), which decreased when blocking I_CaL_ and I_NaL_ (− 82% and − 14%, with 60% I_CaL_ and I_NaL_ block, respectively), and highly increased when blocking I_NCX_ (+ 105% with 40% I_NCX_ block). Therefore, both I_NaL_ and I_CaL_ blocks have a negative effect on CaT_amp_, already reduced in HCM, while I_NCX_ block seems to counteract this aspect of HCM remodelling.

To summarise our findings, I_CaL_ block was identified as the most effective strategy to suppress repolarisation abnormalities in HCM, as well as to revert the increase in APD and [Ca^2 +^]_i,dias_ induced by HCM remodelling. However, large I_CaL_ inhibition may severely compromise CaT_amp_. I_NaL_ block is able to successfully suppress single EADs, but has a very low efficacy on multiple EADs and RF. It also has a positive effect by reducing both APD and [Ca^2 +^]_i,dias_, with a small impact on CaT_amp_. Finally, I_NCX_ block reduces repolarisation abnormalities, especially single EADs, and also shortens APD. However, it has a high impact on CaT, especially on CaT_amp_, and further increases the already elevated [Ca^2 +^]_i,dias_ observed in human HCM cells.

### 3.4 Na^+^/Ca^2 +^ exchanger block improves the efficacy of I_NaL_ and I_CaL_ selective blocks

Simulation results for selective inward current blocks pointed out CaT_amp_ decrease as the main drawback when blocking I_CaL_ and I_NaL_. Since I_NCX_ block produces instead an increase in CaT_amp_, the potential benefit of multichannel block therapies were additionally investigated, by adding I_NCX_ blockade to both the selective I_CaL_ and I_NaL_ blocks. Due to the high number of possible combinations and number of models within the human HCM population (n > 9000), results are presented for a double multichannel block strategy for each pair of currents, based on the findings on selective current block results presented in the previous section. As for I_CaL_, we considered the 40% current block, which yields positive efficacy (100/98% for single/multiple EADs) without excessively compromising the CaT amplitude. As for I_NaL_, as CaT_amp_ reduction was overall of small magnitude, we considered the maximum block (60%). We combined these two scenarios with I_NCX_ block (40% and 20%, respectively), aiming at balancing the changes in both CaT_amp_ and [Ca^2 +^]_i,dias_.

**Fig. 6:**
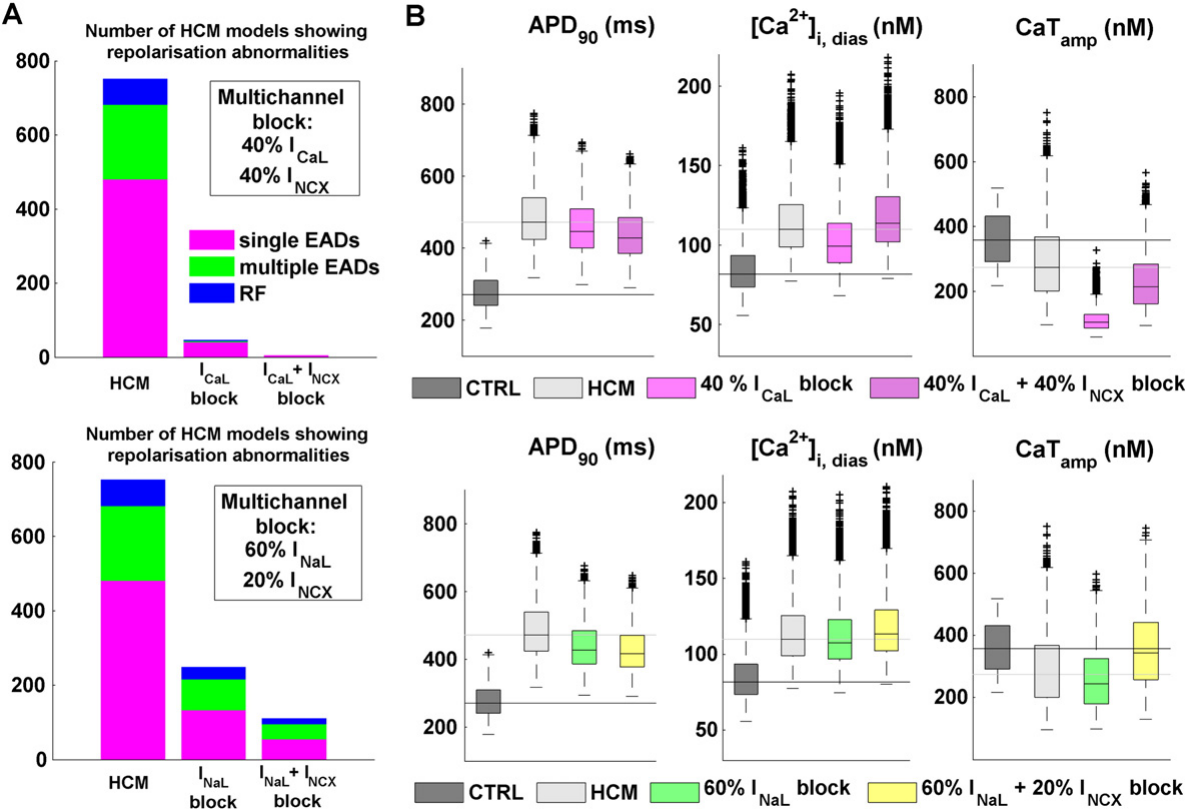
*In silico* evaluation of combined inward channel blocks in human HCM. Simulation results are shown for two different multichannel alternatives: I_CaL_ + I_NCX_ (top row), and I_NaL_ + I_NCX_ (bottom row). A: Efficacy of multichannel strategies in suppressing repolarisation abnormalities, compared to their respective selective current blocks. Bar plots illustrate the number of models exhibiting single/multiple EADs and RF in each considered scenario. B: Effects of multichannel block in reversing APD_90_, [Ca^2 +^]_i,dias_ and CaT_amp_ changes in the human HCM phenotype. CTRL and HCM reference values are also presented to facilitate comparison. Boxplot descriptions as in Fig. 3.

Both multichannel approaches increased the efficacy of the single channel blocks in abolishing repolarisation abnormalities, including single/multiple EADs and RF. Blockade of 40% and 20% of I_NCX_ augmented the efficacy of I_CaL_ and I_NaL_ blocks in + 5% and + 18%, respectively (Fig. 6A). In terms of the normalisation of AP biomarkers, I_NCX_ block further contributed to AP shortening in both multichannel combinations (Fig. 6B, first column), even under already significant APD decrease due to large I_NaL_ inhibition. As for [Ca^2 +^]_i,dias_ (Fig. 6B, second column), the increase in diastolic Ca^2 +^ load induced by I_NCX_ block was not fully compensated by the other single channel block actions: as a result, [Ca^2 +^]_i,dias_ distributions were comparable to those in the baseline HCM population. As for the CaT amplitude (Fig. 6B, last column), the large reduction in CaT_amp_ due to I_CaL_ block was not fully counteracted by additional I_NCX_ block: the final CaT_amp_ was still ~ 100 nM lower than for the HCM population without any current block. On the contrary, when combining I_NaL_ and I_NCX_, the final CaT_amp_ was increased, and almost restored to that for the non-diseased population.

## 4 Discussion

In this study, we unravel the key ionic mechanisms underlying repolarization and Ca^2 +^ handling abnormalities in human ventricular cardiomyocytes in HCM, and we identify potentially-efficacious anti-arrhythmic strategies specific to the HCM phenotype. Our synergistic approach tightly couples populations of models of human ventricular electrophysiology with a rich experimental dataset, including action potential, calcium transient, ionic current and mRNA measurements obtained in human HCM cardiomyocytes. We consistently found the re-activation of the overexpressed L-type Ca^2 +^ current, favoured by a decreased repolarisation reserve, as the key mechanism responsible for repolarisation abnormalities in HCM. These findings were subsequently integrated in *in silico* studies of single and multichannel ion block, aiming at suggesting potential strategies to ameliorate the main hallmarks of the electrophysiological phenotype of human HCM. In spite of exhibiting a high efficacy in the suppression of pro-arrhythmic abnormalities, selective I_CaL_ block also markedly compromised Ca^2 +^ transient amplitude in human HCM. Multichannel I_NCX_, I_NaL_ and I_CaL_ blocks, previously unexplored in the pharmacological management of the disease, exhibited increased efficacy than the respective single blocks, also mitigating adverse effects on systolic Ca^2 +^ function.

In order to address the significant inter-subject variability and disease expression exhibited in the disease, experimentally-calibrated populations of models of human ventricular electrophysiology under non-diseased and HCM conditions were constructed based on the most comprehensive electrophysiological and molecular characterisation of HCM to date in human [3]. The two populations qualitatively and quantitatively reproduce the distinctive characteristics of the non-diseased and HCM phenotypes, and in particular the distinctive characteristics associated to human HCM, including the drastic prolongation of AP duration at different levels of cellular repolarisation, increased diastolic Ca^2 +^ load and decreased Ca^2 +^ transient amplitude with slower kinetics, together with a propensity to develop repolarisation abnormalities.

The population of models approach [17], [31], [32], [33] is particularly suitable for exploring the effect of inter-subject variability in the disease, especially when evaluating potential anti-arrhythmic therapies, whose efficacy may depend on individual responses to drug actions [34]. Furthermore, this is the first time that this methodology has been applied to the study of a genetic cardiomyopathy.

Here, instead of a single AP model representative of average cell behaviour, cell-to-cell differences are accounted for by varying ion channel parameters around their nominal values. These parameters are the maximal conductances of the main ionic currents, based on the assumption that variability is mostly depending on the difference in the number of ionic channels from cell to cell, as recently suggested also by others [19], [34], [35], [36]. Calibration of model outputs against experimental data is then performed in order to only retain models within physiological range. In this regard, each model hence becomes a representation of a viable cell within the plausible bounds of biological variability observed in the population [17], [31], [32], [33].

The HCM phenotype was recovered in this study based on the available experimental data, by modifying the conductances of the main ionic channel affected by the disease together with the inactivation of the L-type Ca^2 +^ current. Additional elements of the electrophysiological remodelling associated to HCM can be easily accommodated to the presented approach, shall the experimental evidence becomes available.

The experimental recordings also provided confirmation of increased proneness to repolarisation abnormalities in HCM diseased cardiomyocytes [3], [37], and in particular to the development of EADs as a well-established pro-arrhythmic mechanism [12], [13]. Our findings suggest that the concomitant decrease of the K^+^ repolarising currents and the increase of inward currents during the plateau phase of the AP, i.e. an impaired repolarisation reserve [30], is the provenance of such repolarisation abnormalities in human HCM. The marked AP prolongation induced by the combined action of these two factors keeps the membrane depolarised long enough to allow for the re-opening of the L-type Ca^2 +^ channels. The mechanistic insights on EADs generation presented in this contribution, specific of human ventricular electrophysiology under HCM diseased conditions, are therefore in agreement with previous experimental and theoretical studies in different animal species [38], [39]. Other possible factors, such as the reopening of Na^+^ channels, has been also associated with EADs triggering [12], [40]. Our *in silico* results, analysed in ~ 800 human ventricular models exhibiting different severities of repolarisation abnormalities, consistently identified I_CaL_ re-activation as the only source of triggered activity in human HCM.

Emerging from these results, we identified the targeting of net inward currents (I_CaL_, I_NaL_ and I_NCX_) as potential therapeutic strategies to counteract AP prolongation in HCM. We evaluated *in silico* their selective block, considering both their anti-arrhythmic implications and their contribution in reversing the HCM phenotype. The selective block of I_CaL_ proved as highly effective in suppressing all repolarisation abnormalities, and also in reducing APD and lowering the diastolic Ca^2 +^ load. However, our simulation results also show how I_CaL_ block markedly affects the already compromised Ca^2 +^ transient amplitude in human HCM (Fig. 5). This provides additional mechanistic and quantitative insights into the interpretation of the existing guidelines for the management of HCM [41], which suggest caution in the use of Ca^2 +^ blockers (e.g. verapamil) in the treatment of the disease [5].

Selective I_NaL_ block successfully suppressed single EADs at high levels of current block, but exhibited small efficacy on more severe repolarisation abnormalities. It also contributed to a decrease in APD and to a smaller extent in diastolic Ca^2 +^ load, without any noticeable effect on Ca^2 +^ transient amplitude. Our findings are hence in agreement with the reported reduction of EADs in HCM cells and the partial reversal of the HCM phenotype under pharmacological I_NaL_ inhibition with ranolazine [3], [42]. In addition, highly selective I_NaL_ blockers are currently being developed and may be available in the near future [43], which together with ongoing clinical trials to assess the effects of ranolazine in HCM patients, will allow for a systematic validation of our prospective *in silico* predictions.

In the HCM population, selective I_NCX_ block had an anti-arrhythmic effect, especially by reducing single EADs and shortening the AP. These results are in agreement with the reported potential of I_NCX_ block in suppressing EADs and preventing Ca^2 +^ overload-induced triggered arrhythmias in canine and guinea pig studies [44], [45], [46]. To facilitate the interpretation of our results, Table 1 summarises the ionic currents investigated in our selective channel block simulation study, drugs targeting them, and their previously reported use in human HCM.

Our *in silico* predictions of combined multichannel I_NCX_ and either I_NaL_ or I_CaL_ blocks in HCM indicate an increase in the efficacy of suppression of repolarisation abnormalities and of the shortening of the AP with respect to the individual selective strategies. On the other hand, our results also show that I_NCX_ block may have a significant impact on Ca^2 +^ handling, by increasing the Ca^2 +^ transient amplitude and further contributing to diastolic Ca^2 +^ overload. These findings are hence supported by those of Bourgonje et al. [28], showing a combined I_CaL_ and I_NCX_ block by SEA-0400 as an effective anti-arrhythmic strategy against dofetilide-induced arrhythmias, despite an increase in diastolic calcium content. Their results were however obtained in a canine chronic atrioventricular block model for compensated hypertrophy, not representative of the ionic remodelling of human HCM. Our study therefore constitutes the first investigation of a multichannel I_NCX_ strategy for the pharmacological management of human HCM, where the arrhythmic mechanisms may be different.

In HCM myocardium, alterations of Ca^2 +^ handling (e.g. reduced SERCA function) may render the cardiomyocyte more dependent on I_NCX_ for Ca^2 +^ transient decay. Additionally, the increased forward mode activity of the Na^+^/Ca^2 +^ exchanger appears to be crucial to maintain CaT amplitude in HCM. For these reasons, the effects of I_NCX_ blockers are likely to be more pronounced in HCM as compared with healthy myocardium. In the multichannel strategies, and under the premise of not significantly raising the diastolic Ca^2 +^ levels observed in HCM, I_NCX_ block only partially ameliorates the drastic decrease of Ca^2 +^ transient amplitude induced by I_CaL_ blockade, whereas when combined with I_NaL_ block it successfully reverts Ca^2 +^ transient amplitude to the values exhibited by the non-diseased population. However, recent experimental findings using last-generation selective I_NCX_ blockers [44], [45], [46] have reported no significant alterations in the AP or Ca^2 +^ transient, in spite of I_NCX_ being considered the main responsible of Ca^2 +^ extrusion in cardiac myocytes. Future research will be devoted to confirm the potential anti-arrhythmic implications of the proposed multichannel strategies for the treatment of human HCM identified in our computational studies.

A different approach to counteract for repolarisation abnormalities in human HCM, not considered in this study, could be the increase of repolarising currents in HCM cardiomyocytes. One possibility could be the use of I_Kr_ agonists to selectively activate these channels, which *in vivo* guinea pig studies with pharmacologically induced QT prolongation successfully shortened the QTc interval [47]. However, additional studies showed that these I_Kr_ agonists also impair cardiac conduction, thus impeding their use as anti-arrhythmic drugs [47]. An alternative option might be the use of ATP-sensitive K^+^ channel openers, as already suggested for heart failure [48]. However, the increased energy requirements of myosin ATPase and the reduction in the phosphocreatine-to-ATP ratio (an established marker of cellular energy status) in HCM [6], [8] may also compromise the availability of these channels for targeting the disease.

Finally, we did not explore the role of beta-adrenergic stimulation in modulating pro-arrhythmic abnormalities in human HCM, whose ionic characterisation still remains unknown in the disease. This may be an important factor of arrhythmic risk in HCM, due to the known effect of autonomic control in modulating ionic currents, and in particular L-type Ca^2 +^ channels [49]. Increased intracellular Na^+^ and Ca^2 +^ levels can also modulate mitochondrial activity linking ATP production to ATP demand, by activation of Na^+^/Ca^2 +^ exchange in the inner mitochondrial membrane, which keeps mitochondrial Ca^2 +^ low preventing ATP supply meeting demand [50]. Computational models of the role of the beta-adrenergic cascade on ionic currents, as well as of mitochondrial ATP synthesis, have been recently proposed [49], [51], [52], [53]. These constitute exciting and promising venues for future investigations.

## Disclosures

None.

## Figures and Tables

**Table 1:** Ionic currents investigated in this study, drugs targeting them, and their previously reported use in the pharmacological management of human HCM.

Compound	Ref	Dose (μM)	Main channel block	Other actions	Use in HCM
Ranolazine	[43]	17 μM	50% I_NaL_	I_Kr_ block	[3], [42]
GS967	[43]	0.13 μM	50% I_NaL_	–	–
Verapamil	[54]	0.33 μM	50% I_CaL_	I_Kr_ block	[5], [41]
ORM-10,103	[44]	0.96 μM	50% I_NCX_	–	–
SEA-0400	[55]	1 μM	66% I_NCX_	I_CaL_ block	–
