# Supplementary material for: Mechanisms of pro-arrhythmic abnormalities in ventricular repolarisation and anti-arrhythmic therapies in human hypertrophic cardiomyopathy
Source: J Mol Cell Cardiol. 2016 Jul;96:72–81. doi: 10.1016/j.yjmcc.2015.09.003 (PMC4915817; doi:10.1016/j.yjmcc.2015.09.003)
Supplement: Supplementary file 1 — Supplementary material [file mmc1.docx]

**Mechanisms of pro-arrhythmic abnormalities in ventricular repolarisation and anti-arrhythmic therapies in human hypertrophic cardiomyopathy**

# SUPPLEMENTARY MATERIAL

**EXTENDED METHODS**

**Modifications to the baseline O’Hara-Rudy human ventricular model**

As baseline for our investigations, a modified version of the endocardial O’Hara-Rudy (ORd) human ventricular action potential model was considered [1]. Minor modifications were performed to the original model to better reproduce the experimental non-diseased data considered in this study, as highlighted in the left panel of Figure S1.

Briefly, the maximal conductance of the transient outward current (I_to_) was increased 2.5 times, based on the comparison between the experimental and simulated I-V curves for I_to_.

To better match the experimental data, we set the extracellular ionic concentrations in the model, as well as the initial values of the intracellular concentrations, to their equivalent experimental solutions in the action potential and calcium transient recordings. This was with the exception of the extracellular K^+^ concentration, as the experimental data used to build the ORd model agree with our data. These modifications in ionic concentrations were not capable to account for the +10 mV shift in resting membrane potential and in the K^+^ equilibrium potential observed in our data compared to the ORd model. Due to these differences, we fixed the K^+^ equilibrium potential to the one experimentally measured for I_K1_. To preserve consistency with the original ORd model, the activation/inactivation curves for I_Na_, I_NaL_ and I_K1_ were shifted by the difference between the original and the modified K^+^ reversal potential (-9.4 mV).

Following the original authors’ recommendation to prevent propagation failure in the model (see http://www.ploscompbiol.org/annotation/listThread.action?root=55207), we modified the steady state inactivation and recovery from inactivation gates for the fast Na^+^ current (h_ss_ and j_ss_). The suggested solution was to replace the I_Na_ formulation with that from Ten Tusscher *et al.* (TT04, [2]). However, the TT04 formulation does not include phosphorylation, which plays an important role in HCM. Therefore, we kept the original ORd formulation and optimised the channel’s steady state inactivation response (half potential and slope) to match that of TT04, using Matlab’s built-in function *fminsearch* (Figure S1, right panel). As for the phosporylated gates, we just applied a -6.2 mV shift in their half potential of inactivation, following the original ORd model based on the work by Wagner *et al.* [3].

Finally, the current stimulus duration was set to 1 ms, with amplitude twice the diastolic threshold, to better match experimental conditions. All model modifications are summarised in Table S1.


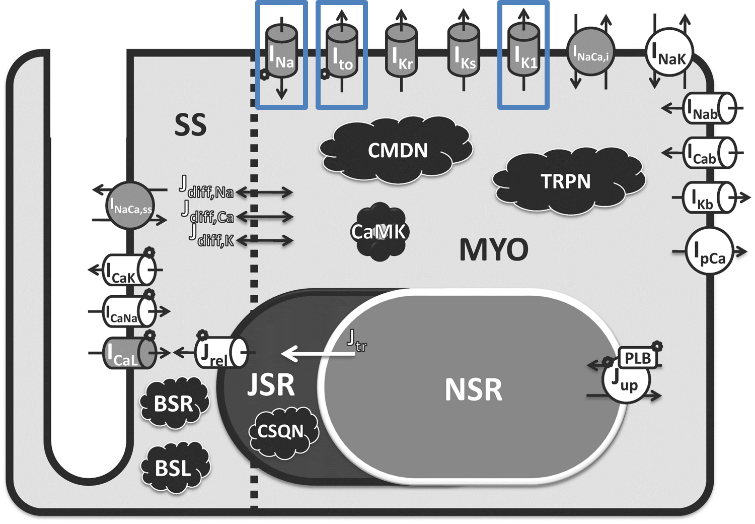
 **
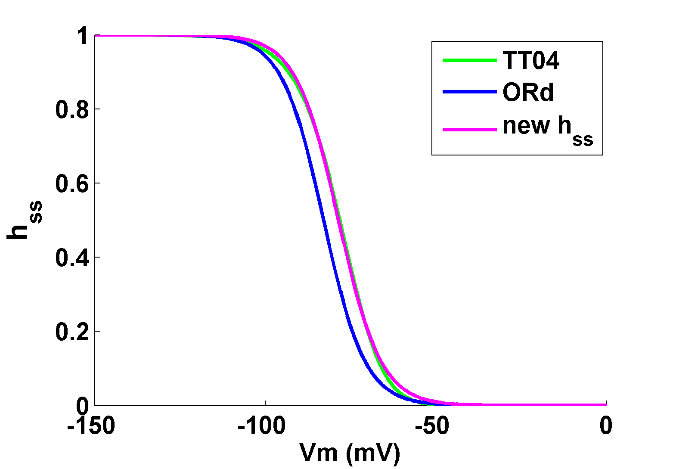
**

**Figure S1.** ***Left:*** Schematic diagram of the O’Hara-Rudy ventricular AP model (modified from [1]). Currents which have been changed in this study are highlighted in blue. ***Right:*** Optimised inactivation and recovery from inactivation gates of the fast Na^+^ current, compared with the original ORd and TT04 formulations.

| **Modification** | **Original ORd formulation** | **Baseline Model formulation** |
| --- | --- | --- |
| I_to_ conductance | $G_{to}=0.02 mS/\mu F$ | $G_{to}=0.02*2.5=0.05 mS/\mu F$ |
| intra- and extra- cellular  ionic concentrations | $\left[ K^{+} \right]_{i}=145 mM (at t=0)$  $\left[ {Na}^{+} \right]_{o}=140 mM$ | $\left[ K^{+} \right]_{i}=140 mM (at t=0)$  $\left[ {Na}^{+} \right]_{o}=132 mM$ |
| K^+^ equilibrium potential | $E_{K}=(RT/F)*log(\left[ K^{+} \right]_{o}/\left[ K^{+} \right]_{i})$ | $E_{K}=-77.6 mV$  I_Na_, I_NaL_, I_K1_ activation/inactivation gates shifted of -9.4 mV |
| I_Na_ steady state inactivation and recovery gates (h and j) | $h_{ORd}=j_{ORd}= \frac{1}{1+e^{\frac{v+82.9}{6.09}}}$ | $h_{opt}=j_{opt}= \frac{1}{1+e^{\frac{v+78.5}{6.22}}}$ |
| Current stimulus | $amplitude= -80 \mu A/\mu F$ $duration= 0.5 ms$ | $amplitude= -53 \mu A/\mu F$ $duration= 1 ms$ |

**Table S1.** Comparison between the original ORd model and the baseline model used in this study.

**Electrophysiological remodelling in HCM vs heart failure**

The electrophysiological remodelling considered in this study to reproduce the HCM phenotype is compared below against the most recent simulation studies on heart failure (HF). A summary of the percentual changes for each ionic current is presented in Table S2. All numerical values have been computed from experimental data, as detailed in the cited studies and references therein.

| **Ionic Current** | **HCM** | **HF** | **Ionic Current** | **HCM** | **HF** |
| --- | --- | --- | --- | --- | --- |
| **I_Na_** | - | -57% [4] | **I_to_** | -70% | -60/73% [4–6] |
| **I_NaL_** | +165% | +80/200% [4,5] | **I_K1_** | -30% | -32/50% [4,5] |
| **I_Nab_** | +165% | - | **I_Kr_** | -45% | -45% [4,6] |
| **I_CaL_** | +40% | 0/-60% [4–6] | **I_Ks_** | -45% | -57% [4] |
| **τ I_CaL_**  *fast inactivation* | +35% | - | **I_NaK_** | -30% | -30/42% [4,5] |
| **τ I_CaL_**  *slow inactivation* | +20% | - | **J_Up_** | -25% | -25/50% [4–6] |
| **I_NCX_** | +30% | +75/200% [4–6] | **J_Rel_** | -20% | -20% [5] |

**Table S2.** Comparison of the ionic electrophysiological remodelling in HCM vs HF.

**EXTENDED RESULTS**

**Sensitivity Analysis**

To investigate the correlation between the parameters varied in the population of models and the action potential and calcium transient biomarkers considered in this study, we performed a sensitivity analysis based on partial correlation methods [7], as illustrated in [8]. We chose to use the partial correlation method over other correlation measures because it allows taking into account the effects of one or more additional variables when looking for correlation between two quantities. This is particularly important when considering populations of models, since they are generated by varying multiple parameters at the same time (n=11 in this study).

The partial correlation coefficient ($PCC$) between two variables ($x$and $y$), given $N$additional variables ($z_{i}$) is computed by first calculating the linear regression models of $x$ and $y$ against $z_{i}$:

$\hat{x}=c_{0}+\sum_{i=1}^{N} ({c_{i}\cdot z}_{i})$ $\hat{y}=b_{0}+\sum_{i=1}^{N} {{(b}_{i}\cdot z}_{i})$

$PCC$ is then defined as the correlation coefficient between the residuals $r_{x}=x-\hat{x}$ and $r_{y}=y-\hat{y}$:

$$PCC\left( x,y,z_{i} \right)=\frac{Cov\left( r_{x},r_{y} \right)}{Var\left( r_{x} \right)\cdot Var{(r}_{y})}$$

Partial correlation coefficients span from -1 to +1, where -1 indicates a strong negative correlation between the two considered variables, and +1 a strong positive one.

The sensitivity analysis results for both the CTRL and HCM populations are shown in Figure S2 (top and bottom panels, respectively). K^+^ repolarising currents (in particular I_Kr_ and I_Ks_) are negatively correlated with APDs, while the inward currents (especially I_NaL_, I_CaL_ and I_NCX_) are positively correlated with them. J_up_ and J_rel_ have a strong influence on Ca^2+^-transient, and I_Na_ is positively correlated with both dV/dt_MEAN_ and AP_amp_. In HCM, where I_NaL_ is highly increased (+265%), the correlation with the biomarkers is stronger.

**
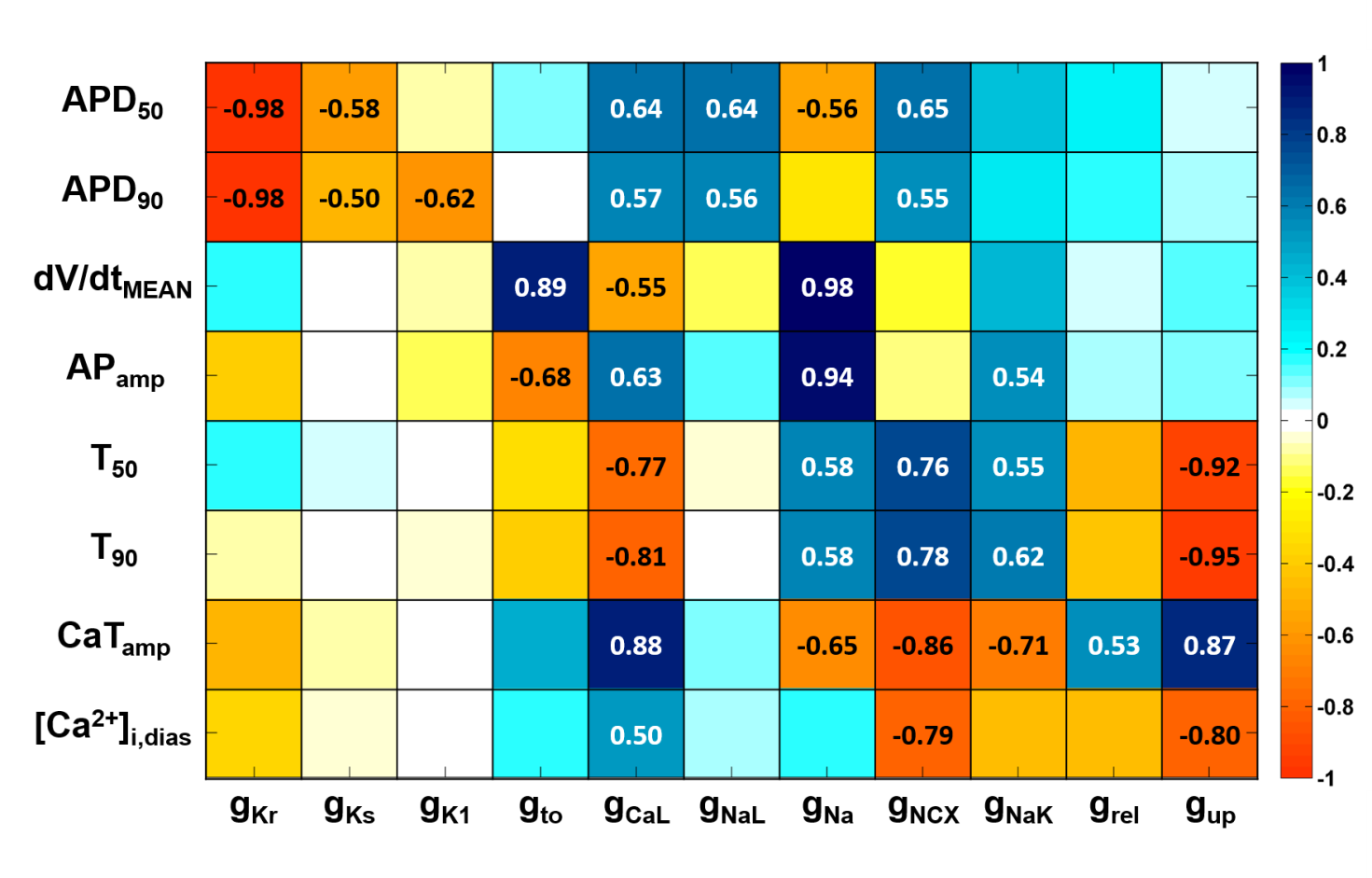

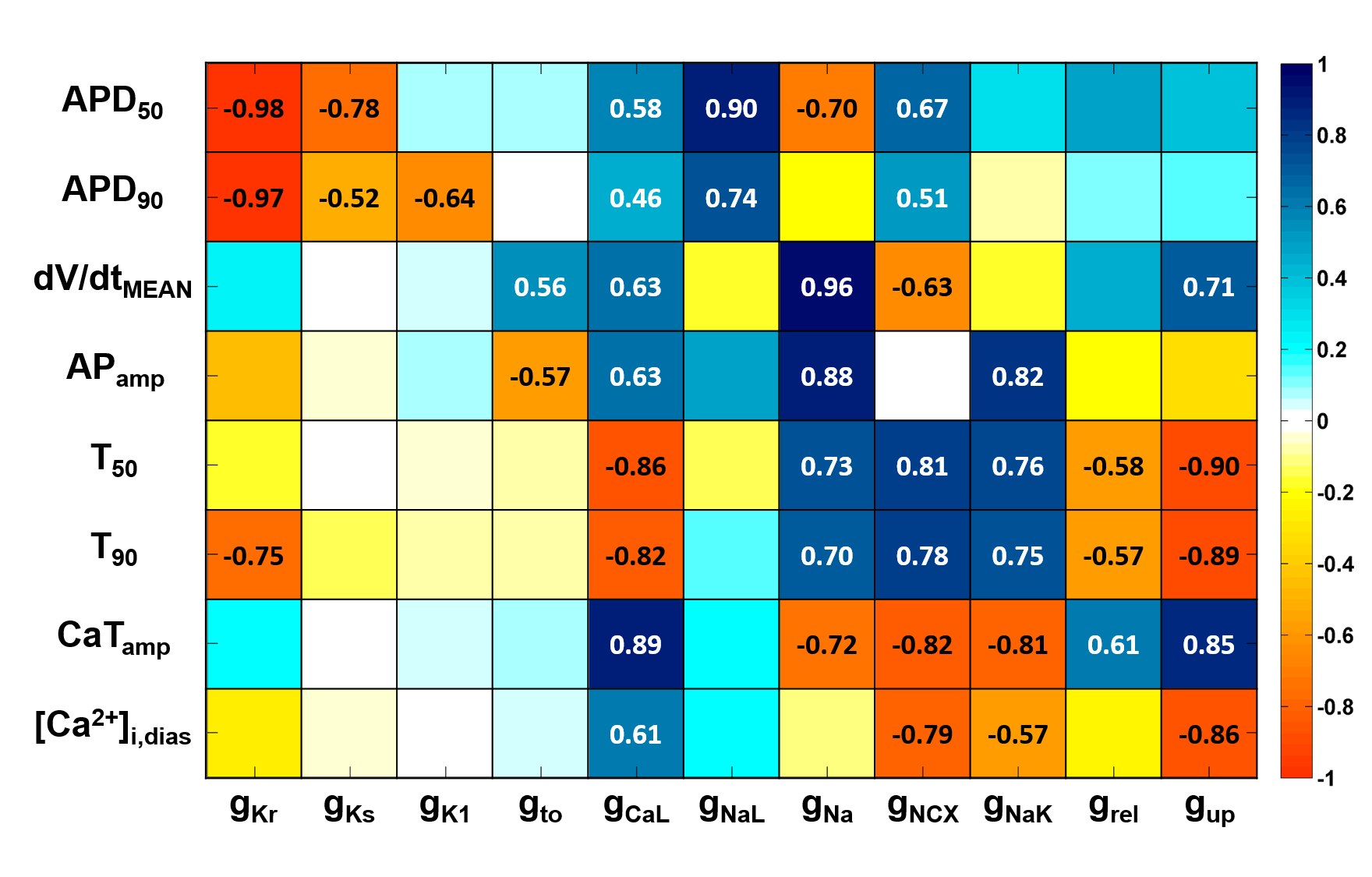
**

**Figure S2.** Sensitivity analysis for the CTRL (top) and HCM (bottom) populations of models, quantifying the correlation between parameters varied in the populations and the AP and CaT biomarkers. The colour scale represents the partial correlation coefficient (PCC). Numerical values are indicated only when $|PCC|\geq0.5$.

**SUPPLEMENTAL REFERENCES**

[1] O’Hara T, Virág L, Varró A, Rudy Y. Simulation of the undiseased human cardiac ventricular action potential: model formulation and experimental validation. PLoS Comput Biol 2011;7:e1002061. doi:10.1371/journal.pcbi.1002061.

[2] Ten Tusscher KHWJ, Noble D, Noble PJ, Panfilov A V. A model for human ventricular tissue. Am J Physiol Heart Circ Physiol 2004;286:H1573–89. doi:10.1152/ajpheart.00794.2003.

[3] Wagner S, Dybkova N, Rasenack ECL, Jacobshagen C, Fabritz L, Kirchhof P, et al. Ca2+/calmodulin-dependent protein kinase II regulates cardiac Na+ channels. J Clin Invest 2006;116:3127–38. doi:10.1172/JCI26620.

[4] Elshrif MM, Pengcheng Shi, Cherry EM. Electrophysiological properties under heart failure conditions in a human ventricular cell: a modeling study. Conf Proc. Annu Int Conf IEEE Eng Med Biol Soc IEEE Eng Med Biol Soc Annu Conf 2014;2014:4324–9. doi:10.1109/EMBC.2014.6944581.

[5] Gomez JF, Cardona K, Romero L, Ferrero JM, Trenor B. Electrophysiological and structural remodeling in heart failure modulate arrhythmogenesis. 1D simulation study. PLoS One 2014;9:e106602. doi:10.1371/journal.pone.0106602.

[6] Walmsley J, Rodriguez JF, Mirams GR, Burrage K, Efimov IR, Rodriguez B. mRNA expression levels in failing human hearts predict cellular electrophysiological remodeling: a population-based simulation study. PLoS One 2013;8:e56359. doi:10.1371/journal.pone.0056359.

[7] Marino S, Hogue IB, Ray CJ, Kirschner DE. A methodology for performing global uncertainty and sensitivity analysis in systems biology. J Theor Biol 2008;254:178–96. doi:10.1016/j.jtbi.2008.04.011.

[8] Britton OJ, Bueno-Orovio A, Van Ammel K, Lu HR, Towart R, Gallacher DJ, et al. Experimentally calibrated population of models predicts and explains intersubject variability in cardiac cellular electrophysiology. Proc Natl Acad Sci U S A 2013;110:E2098–105. doi:10.1073/pnas.1304382110.
